# New lipases by mining of *Pleurotus ostreatus* genome

**DOI:** 10.1371/journal.pone.0185377

**Published:** 2017-09-25

**Authors:** Alessandra Piscitelli, Vincenzo Tarallo, Lucia Guarino, Giovanni Sannia, Leyla Birolo, Cinzia Pezzella

**Affiliations:** Dipartimento di Scienze Chimiche, Università degli Studi di Napoli Federico II, Napoli, Italy; Leibniz-Institut fur Pflanzengenetik und Kulturpflanzenforschung Gatersleben, GERMANY

## Abstract

The analysis of *Pleurotus ostreatus* genome reveals the presence of automatically annotated 53 lipase and 34 carboxylesterase putative coding-genes. Since no biochemical or physiological data are available so far, a functional approach was applied to identify lipases from *P*. *ostreatus*. In the tested growth conditions, four lipases were found expressed, with different patterns depending on the used C source. Two of the four identified proteins (PleoLip241 and PleoLip369), expressed in both analysed conditions, were chosen for further studies, such as an *in silico* analysis and their molecular characterization. To overcome limits linked to native production, a recombinant expression approach in the yeast *Pichia pastoris* was applied. Different expression levels were obtained: PleoLip241 reached a maximum activity of 4000 U/L, whereas PleoLip369 reached a maximum activity of 700 U/L. Despite their sequence similarity, these enzymes exhibited different substrate specificity and diverse stability at pH, temperature, and presence of metals, detergents and organic solvents. The obtained data allowed classifying PleoLip241 as belonging to the “true lipase” family. Indeed, by phylogenetic analysis the two proteins fall in different clusters. PleoLip241 was used to remove the hydrophobic layer from wool surface in order to improve its dyeability. The encouraging results obtained with lipase treated wool led to forecast PleoLip241 applicability in this field.

## Introduction

Lipolytic enzymes are grouped into two major families, lipases (EC 3.1.1.3) and carboxylesterases (EC 3.1.1.1). Lipolytic enzymes are able to hydrolyze or synthesize ester bonds, depending on the amount of water in the reaction medium [1]. Lipolytic enzymes have a highly conserved catalytic triad, composed of Ser, Asp, and His residues [2]. The catalytic serine is part of a conserved pentapeptide (G-X-S-X-G) [3]. These enzymes belong to the α/β hydrolase family with a central β-sheet, containing the active serine placed in a loop termed the catalytic elbow [4]. Although distinction between lipases and carboxylesterases is still a matter of debate, one of the criteria of classification was based on substrate specificity. True lipases attack triacylglycerols with fatty acid chain lengths of more than C10 [5], whereas esterases hydrolyse glycerolesters with fatty acid chain lengths of C10 or shorter chains. The biological function of lipases is the hydrolysis of triacylglycerols, however they also display alcoholysis, aminolysis, interesterification, and esterification activity, with rigorous regioselectivity, stereoselectivity, and chemoselectivity. Lipases are produced by animals, plants, and microorganisms including bacteria, yeast, and fungi [6]. Over the last few years, there has been a progressive increase in the number of publications related to proprieties and industrial applications of microbial lipases in several sectors, ranging from food, pharmaceutical, cosmetic, leather, biofuel, laundry, and in several bioremediation processes. In this context it is understandable that reducing production costs at large scale industrial level, and discovering new strains for the production of novel lipases with industrially useful properties, is increasingly becoming a new interest area of lipase research [7,8].

Only few examples of lipolytic enzymes from basidiomycetes fungi are present in the recent literature [9–15]. However, lipases have never been isolated from the basidiomycete fungus *Pleurotus ostreatus* even if many putative lipase coding genes have been automatically annotated in its genome [16,17]. On the other hand, enormous efforts have been made to characterize its oxidative enzymatic systems [18,19].

Considering that no biochemical or physiological data about *P*. *ostreatus* lipolytic enzymes are available, in this study several culture conditions were explored to verify the functional expression of lipolytic enzymes from this fungus. Lipase production was verified in different conditions and the produced enzymes identified and characterized. Selected enzymes were further studied by means of an *in silico* analysis and molecular characterization. Based on substrate specificity, one of the enzymes was classified as true lipase. Capability to improve dyeability of wool was tested, demonstrating a possible application in the textile field.

## Materials and methods

### Materials

Reagents were purchased from Sigma-Aldrich Corp. (St. Louis, MO). The expression vector pJGGαKR was purchased from Biogrammatics, Ltd (Las Palmas Dr, Carlsbad, CA, USA). DNA restriction and modifying enzymes were supplied by Promega (Madison, WI, USA). Culture media were bought from BD Difco (Becton Drive, Franklin Lakes, NJ USA).

### *P*. *ostreatus* culture conditions

The basidiomycete fungus used in this study was *P*. *ostreatus* (Jacq.:Fr.) Kummer (type: Florida) (ATCC MYA-2306) from ATCC, the Global Bioresource Centre. The fungus was maintained through periodic transfer at 4°C on PDY broth (24 g/L potato dextrose and 5g/L yeast extract).

To detect lipase activity on chromogenic plates, fungus mycelium (a 5 mm diameter agar plug from the edge of a 7 day old agar culture) was grown on PDY agar plates (diameter 100 mm) supplemented with 1% olive oil, 1% phenol red and 1% CaCl_2_, and incubated in the dark for 4 days at 28°C and checked for the development of yellowish halo indicating the presence of lipolytic activity. The effect of the following carbon sources on lipase secretion was evaluated: olive oil (0.1–1%), Olive Mill Wastewater (OMW) (1–10%), glycerol (0.1–1%), glucose (5–10 g/L).

*P*. *ostreatus* pre-cultures were prepared as follows: 250 Erlenmeyer flasks containing 75mL of PDY broth. Flasks were maintained in continuous agitation at 125 rpm and 28°C in complete darkness. After 5 days, the entire culture was homogenized by Waring Blender 7011HS^®^ (3 flashes of 3 sec at maximum rpm) and inoculated in a 1:10 ratio in PDY broth added with the carbon sources allowing lipase production, at the lowest concentrations: 1% olive oil, 5% OMW, 10 g/L glucose.

### Lipase assay

Lipase activity was determined spectrophotometrically (UVIKON 922 UV/Vis Spectrophotometer, BioTek Instruments) using *p*-nitrophenyl decanoate as substrate. The *p*-nitrophenyl decanoate was dissolved in isopropanol at a concentration of 10 mM. The assay was carried in 50 mM Tris-HCl (pH 8.0) with 0.2 mM the *p*-nitrophenyl decanoate. The activity was assayed by detecting the released product, *p*-nitrophenol, at 405 nm (ε_405_ = 3,390 M^−1^cm^−1^).

### Polyacrylamide gel electrophoresis (PAGE)

Native PAGE was performed at alkaline pH. The separating and stacking gels contained, respectively, 9% and 4% acrylamide and 50 mM Tris-HCl (pH 9.5) and 18 mM Tris-HCl (pH 7.5) as buffers. The electrode reservoir solution contained 25 mM Tris and 190 mM glycine (pH 8.4). After electrophoresis the gel was rinsed in dH_2_O and in 20 mM Tris-HCl (pH 8). Visualization of the bands was achieved by overlapping to the gel a thin layer of polymerized agarose containing 1% olive oil, 1% phenol red, 1% CaCl_2_.

The purity of the sample and the Mw of the protein were verified by 15% SDS-PAGE, stained with Coomassie brilliant blue R-250. The molecular weight standard used was PageRuler^™^ Plus Prestained Protein Ladder (200–10 kDa) from ThermoFisher Scientific.

### Protein identification

Proteins were identified by standard proteomic strategies on gel bands exhibiting enzymatic activity, following the procedures as already reported [20]. Briefly, bands corresponding to active proteins were excised from the gel and repeatedly washed with acetonitrile and 0.1 M ammonium bicarbonate. Cysteines were reduced with 10 mM dithiothreitol (DTT) for 45 min at 56°C and alkylated by incubation in 5mM iodoacetamide for 15 min at room temperature in the dark. Enzymatic digestion was carried out with trypsin (12.5 ng/μL) in 50mM ammonium bicarbonate buffer, pH 8.5, at 4°C for 2h. a new aliquot of buffer solution with trypsin was added and the sample incubated for 18h at 37°C. Peptides were extracted with 0.1% (v/v) formic acid in 50% (v/v) acetonitrile at room temperature and lyophilized. Peptide mixtures were analyzed by LC–MS/MS, on a HPLC–Chip LC system (Agilent 1200) connected to a Q-TOF 6520 (Agilent Technologies). Lyophilized samples were resuspended in 10 μL of 0.1% (v/v) formic acid. After loading, the peptide mixtures were concentrated and washed at 4 μL/min in a 40 nL enrichment column with 0.2% (v/v) formic acid in 2% (v/v) acetonitrile. Fractionation was carried out on a C-18 reverse phase column (75μm×43mm) at a flow rate of 0.4μL/min with a linear gradient of eluent B (95% v/v acetonitrile and 0.2% v/v formic acid) in eluent A (2% v/v acetonitrile and 0.1% v/v formic acid) from 7% to 80% in 51 min. Mass spectrometry analyses were performed using data dependent acquisition MS scans (mass range 300–2400m/z), followed by MS/MS scans (mass range 100–2000m/z) of the 4 most intense ions of a chromatographic peak. Raw data from LC–MS/MS were converted to m/z data, and searched against the PleosPC15 database available at the Joint Genome Institute′s website (http://genome.jgi-psf.org/PleosPC15_2) using the licensed version of Mascot 2.1 (Matrix Science).

### Bioinformatics analysis

Protein sequences were aligned with those available in the GenBank database [21] using the Blast software at the National Centre of Biotechnology Information website (http://www.ncbi.nlm.nih.gov) [22]. The 3D structure models were built with Phyre2 program [23]. N-glycosylation sites were identified with NetNGlyc 1.0 server (http://www.cbs.dtu.dk/services/NetNGlyc/).

A Neighbour Joining tree was constructed using MEGA 7.0 software [24] based on the alignment of PleoLip241 and PleoLip369 protein sequences with ClustalW using default settings for multiple sequence alignments.

### Recombinant expression in *P*. *pastoris*

The BG-10 *Pichia pastoris* strain (BioGrammatics Ltd.) was used for the heterologous expression and was propagated in YPDS medium (10 g/L yeast extract; 20 g/L bacto tryptone; 20 g/L glucose; 182.2 g/L sorbitol).

*PleoLip241* and *PleoLip369* coding genes, excluding the signal peptide regions, were optimized according to *P*. *pastoris* codon usage and synthesized (Thermo Fischer Scientific, Waltham, Massachusetts, USA). Genes were hydrolyzed with *Bsa*I and ligated into the corresponding site of the pJGGαkR vector in-frame with the α-factor signal peptide under the control of the constitutive glyceraldehyde-3-phosphate dehydrogenase (*GAP*) promoter, yielding the recombinant pJGGαKR /PleoLip241 and pJGGαKR /PleoLip369 plasmids. Both plasmids were linearized by *Bsi*WI and transformed into *P*. *pastoris* BG10 by electroporation, as already reported [25].

Tributyrin agar plates (5 g/L peptone; 3 g/L yeast extract; 0.1% tributyrin; 20 g/L agar, pH 6.0 [26]) were used to identify the highest-producing *P*. *pastoris* clones after the transformations with pJGGαKR /PleoLip241 and pJGGαKR /PleoLip369 constructs. Plates were incubated upside down for 5 days at 28°C and checked for the appearance of a clear halo. Positive clones were inoculated in liquid media, and daily assayed for cell density and secreted lipase activity.

Selected recombinant clones were inoculated in 50 mL BMGY medium (13 g/L yeast nitrogen base with ammonium sulfate without amminoacids; 10 g/L yeast extract; 20 g/L peptone; 100 mM potassium phosphate, pH 6.0; 4x10^-4^ g/L biotin; 1% glycerol) in a 250 mL baffled shaken flask. This preculture was grown overnight at 28°C on a rotary shaker (250 rpm), then a volume of suspension sufficient to reach a final OD_600_ value of 1.0 was used to inoculate 1 L shaken flasks containing 250 mL of BMGY medium. Cells were grown on a rotary shaker (250 rpm) at 28°C. YP (10 g/L Yeast extract, and 20 g/L peptone) broths added with different carbon sources (10 g/L glucose, 1% glycerol, 2% oleic acid, 2% palm oil) were tested as alternative growth media. The supernatant was daily recovered and assayed for lipase production.

### Protein purification

After the growth, the cells were harvested by centrifugation at 8,000 g at 4°C for 15 min. The sample was concentrated and dialysed on T-Series TFF Cassettes system (10 KDa cutoff membrane) (PALL Corporation). The samples dialysed in 50 mM Tris-HCl pH 7 were loaded on CM Sepharose FF column (GE Healthcare) previously equilibrated in the same buffer. The proteins were eluted with a NaCl gradient (0 to 1 M NaCl) in the same buffer.

### Recombinant protein characterization

#### Effect of pH and temperature on lipase activity and stability

The optimal pH of lipase activity was determined using *p*-nitrophenyl decanoate in the range of 2 to 12 by using different pH buffer solutions (McIlvaine buffer, pH 2–8; 50 mM Tris-HCl, pH 8–10; sodium carbonate, pH 10–12) at room temperature (25°C). The optimal temperature of the enzyme activity was evaluated in the range 30° - 60°C in 50 mM Tris-HCl buffer, pH 8.

pH stability was assessed by incubating the enzyme at the same pHs (2–12). Lipase thermostability was determined by incubating the enzyme in the temperature range 30–60°C.

All the measurements were made in triplicate.

#### Substrate specificity

*p*-nitrophenyl esters containing acyl chains with different length (Acetate, Butyrate, Valerate, Octanoate, Decanoate, Dodecanoate, Myristate, Palmitate, Stearate) were used to determine the substrate specificity. The assays were carried out in standard conditions (50 mM Tris-HCl pH 8, 25°C). All the measurements were made in triplicate.

#### Effect of metal ions, detergents and organic solvents

The effect of metal ions, solvents and detergents on PleoLip241 and PleoLip369 activities after incubation of 4 hours at 25°C was measured. The assays were carried out in standard conditions (50 mM Tris- HCl pH 8, 25°C). All the measurements were made in triplicate.

#### Kinetic parameters determination

Kinetic parameters were measured at pH 8 using *p*-nitrophenyl esters in the range 0.001–1 mM. Kinetic parameters were determined by a non-linear regression curve using GraphPad Prism version 7.00 for Windows, GraphPad Software (http://www.graphpad.com).

### Wool dyeing

Twenty mg of pure new wool were treated with 3U of PleoLip241 in 50 mM Tris-HCl pH 8. The reaction mixture was incubated for 2 hours at 40°C in shaking conditions (100 rpm). The untreated wool was used as control. After treatment, treated and untreated wool were dyed using 10 mM of the commercial dye Direct Blue 71. Dyeing was performed at 60°C for 1 hour in shaking conditions (100 rpm). After the dyeing, the wool was squeezed and the absorbance spectrum of the colouration bath was analysed using an UVIKON 922 UV/Vis Spectrophotometer (BioTek Instruments). The percentage of the dye remaining in the colouration bath was calculated measuring the Abs_594 nm_ of the colouration bath before and after the wool dyeing.

## Results and discussion

### *P*. *ostreatus* lipases

**Fig 1:**
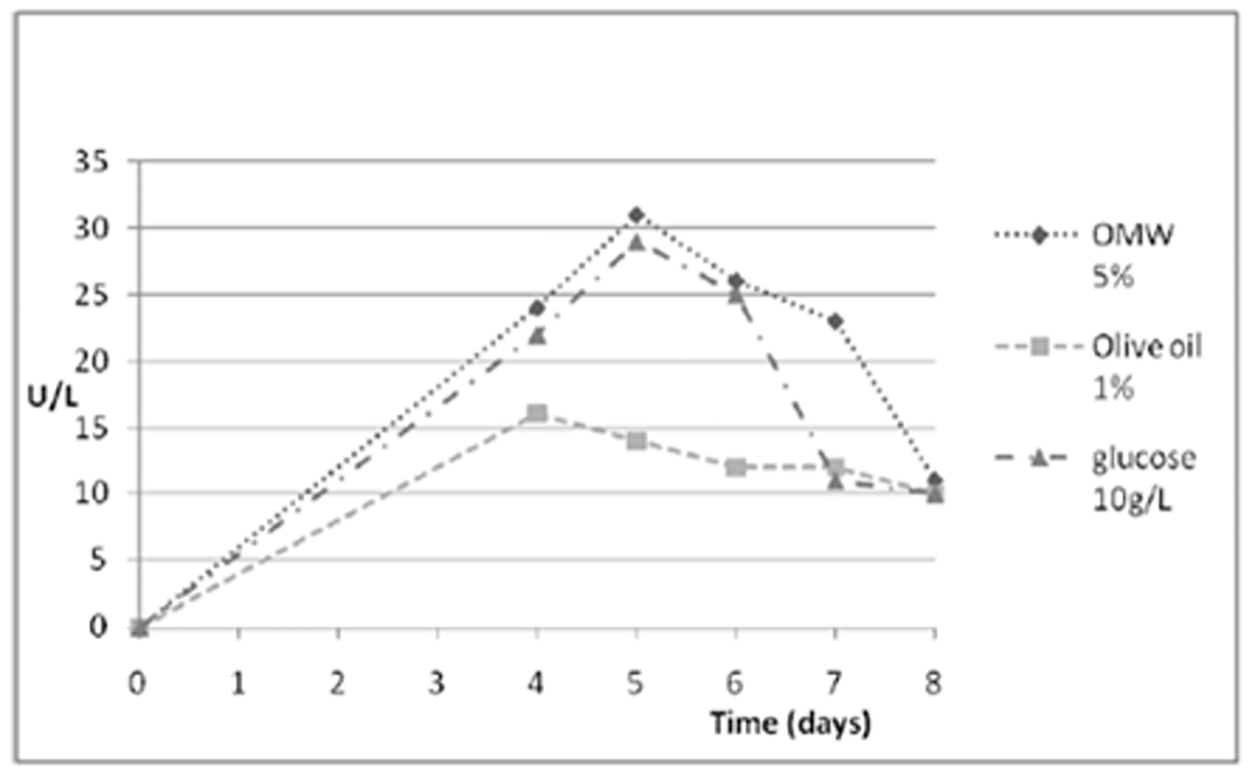
Time course activity. Extracellular lipases produced by *P*.*ostreatus* in different culture conditions. All experiments have been conducted in triplicate. Standard deviations among three replicates were less than 5%.

**Fig 2:**
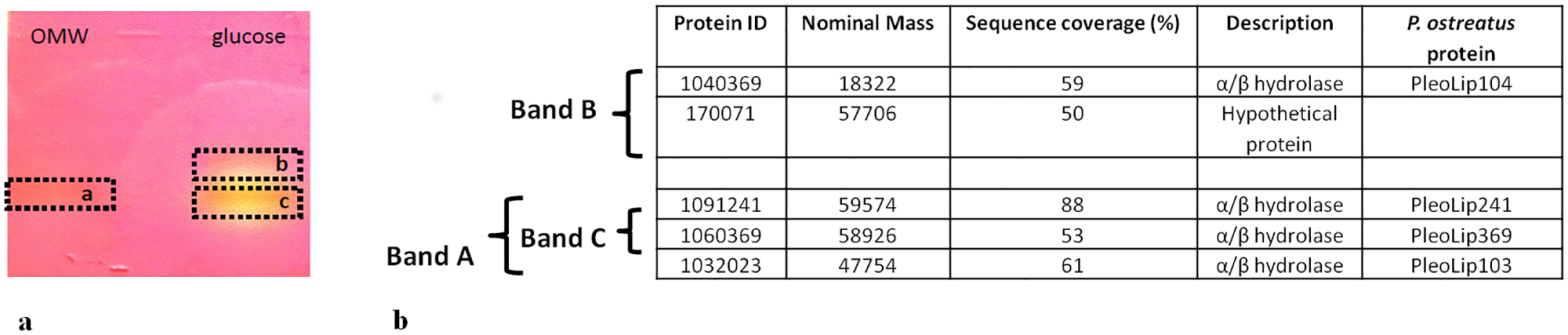
Secreted lipases from *P*. *ostreatus*. **(a)** Native PAGE of the extracellular proteins; **(b)** Protein identification.

An analysis of the *P*. *ostreatus* genome searching for lipolytic coding genes, revealed the presence of 53 putative lipase and 34 putative carboxylesterase coding-genes, with five genes in common between the two groups. Considering that all these genes are postulated to be lipolytic enzymes based on automated annotation, and no biochemical or physiological data are available so far, in this study a functional approach was applied to identify lipases from *P*. *ostreatus*. With the aim to stimulate lipase production from *P*. *ostreatus* and characterise the extracellular enzymes, the fungus was grown in the presence of different carbon sources (olive oil, OMW, glycerol and glucose) (S1 Table). The different conditions were analysed by a chromogenic screening on plate, using olive oil as substrate [27]. The choice of this substrate was aimed at selecting true lipases, such as lipolytic enzymes able to hydrolise acyl glycerols with fatty acid chain lengths of more than C10. All carbon sources, except glycerol, led to the secretion of active lipases. Time course analysis of extracellular lipolytic enzyme production was conducted in selected conditions (1% olive oil, 5% OMW, 10 g/L glucose) (Fig 1). Production profiles in the presence of 5% v/v OMW and 10 g/L glucose were comparable and higher than that observed in the presence of 1% olive oil. A maximum production of about 30 U/L in five days was obtained in the best growth conditions. The obtained value is in the same range of other reports [15]. Proteins secreted in the presence of OMW and glucose were analysed through native PAGE with the aim to verify the number of lipases secreted (Fig 2a). In the presence of OMW only an active lipase band was detectable, while two active lipases were detected in the presence of glucose. Protein bands exhibiting enzymatic activity were identified by LC-MSMS analysis searching the annotated *P*. *ostreatus* genome. Identified proteins are reported in Fig 2b. It is worth to note that in these two selected conditions at least 4 putative lipase coding genes were expressed with a pattern depending on the culture conditions: *PleoLip241*, *PleoLip369*, *PleoLip103* and *PleoLip104*. Two of the four identified proteins (PleoLip241 and PleoLip369), expressed in both analysed conditions, were chosen for further studies, such as an *in silico* analysis and their molecular characterization by recombinant expression.

### Bioinformatic analysis

**Fig 3:**
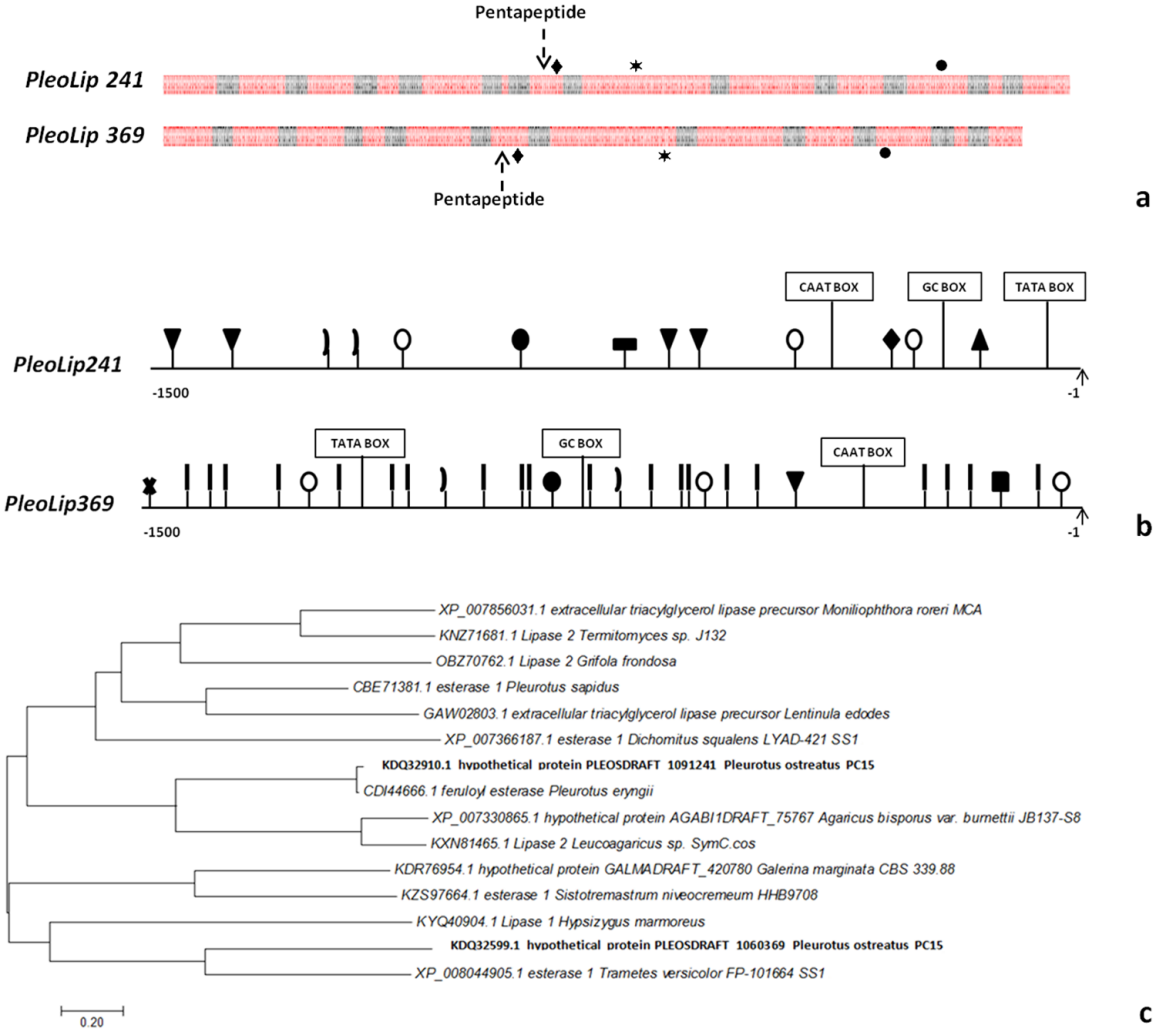
Bioinformatic analysis of *PleoLip241* and *PleoLip369*. **(a)** Gene structure of coding sequences. Red boxes exons, black boxes introns. The sequence coding for the pentapeptide (G-X-S-X-G) and for the three aminoacids of the catalytic triad are shown in the picture: (black diamond) Ser, (black star) Asp, (black circle) His; **(b)** Distribution of putative cis-acting elements in the promoter regions, around 1500 bp upstream of the start codons: TATA box; CAAT box; GC box; (black square) FARE, (black vertical bar) HSE; (black closed parenthesis) NIT2; (upside black triangle) STRE; (black triangle) MRE; (black circle) O2-site; (white circle) PRE; (black rectangle) TGACG-motif; (black cross) CGTCA-motif; **(c)** Neighbour Joining tree of protein sequences. The tree is drawn to scale, with branch lengths in the same units as those of the evolutionary distances used to infer the phylogenetic tree. All positions containing alignment gaps and missing data were eliminated only in pairwise sequence comparisons (pair wise deletion option). Phylogenetic analyses were conducted in MEGA 7.0 [24]. *P*. *ostreatus* lipases are highlighted in bold.

The sequences coding for *PleoLip241* (2,327 bp long) and *PleoLip369* (2,213 bp long) display a 52.6% of identity and are interrupted by twelve and eleven introns, respectively. All the splicing junctions of introns adhere to the GT-AG rule and most of the internal lariat sites conform to the consensus CTRAY [28]. The intron size ranges from 48 to 60 bp, similarly to most of the fungal introns [29]. A common gene structure is shared between the two genes, as displayed in the Fig 3a.

The promoter regions extending 1500 bp upstream of the ATG were searched for consensus sequences: fatty acid responsive elements FARE (CCTCGG) [30]; heat shock elements (HSE, NGAAN) [31]; NIT2 binding site (TATCT) [32] putative response elements PRE (ATATC and TGGGT motifs) [33]; cAMP-response elements (CGTCA and TGACG-motifs) [34,35]; O2-site (GATAA) [36]; Cre-A-binding site (GCGGGG) [37]; and stress-responsive elements (STRE, CCCCT) [38]. Several putative response elements were identified, differentially distributed along the promoter sequences (Fig 3b). STRE, NIT2 and O2-site elements were found in both promoter sequences. In *PleoLip369*, a free fatty acid element (FARE) and a high number of HSE elements were also identified.

PleoLip241 and PleoLip369 proteins display 58.9% of sequence identity and are closely related to other microbial lipases. PleoLip241 shows the highest identity with a lipase from *Pleurotus eryngii* (97.4%), whereas PleoLip369 with a lipase from *Hypsizygus marmoreus* (53%).

The phylogenetic positioning of PleoLip241 and PleoLip369 is shown in Fig 3c. Based on this Neighbor Joining (NJ) tree, PleoLip241 and PleoLip369 fall in different clusters.

A multiple alignment, with lipases whose 3D structures have been determined and with lipases showing the highest identity with the new Pleo-lipases, allowed to identify the residues of the catalytic triad (Ser, Asp, His) and of the conserved pentapeptide (G-X-S-X-G) [3]. In the case of PleoLip241 the conserved residues of the active site are Ser^273^, Asp^403^, His^520^; with GQSAG as pentapeptide. In the case of PleoLip369 the catalytic triad is Ser^266^, Asp^394^, His^512^ and the conserved pentapeptide GESAG (S1 Fig). Four potential N-glycosylation sites can be identified in PleoLip241 (Asn-132, Asn-360, Asn-396, Asn-521) and six in PleoLip369 (Asn-24, Asn-31, Asn-128, Asn-342, Asn-366, Asn-390).

### Recombinant production of PleoLip 241 and PleoLip369

PleoLip 241 and PleoLip369 were expressed in the yeast *P*. *pastoris* under the control of the constitutive *GAP* promoter. Both proteins were found to be secreted in active form in the extracellular broth in the different culture conditions tested. The best results were obtained growing the recombinant yeasts in BMGY at 28°C for both proteins. PleoLip241 production reached a maximum of 4000 U/L, whereas a maximum of 700 U/L was obtained for PleoLip369 after 9 days of growth. Both production levels are very interesting if compared with those reported for the recombinant expression of other basidiomycete lipases. Krugener and co-authors performed a recombinant expression in *Escherichia coli* of a lipase from *Pleurotus sapidus* achieving a production of 116 U/L [9]. The recombinant expression in *P*. *pastoris* of a lipase from *Bjerkandera adusta* led to a production of about 40 U/L [39].

PleoLip 241 and PleoLip369 enzymes were purified to homogeneity (S2 Fig) through an ultrafiltration step followed by a cationic exchange chromatography.

### Molecular characterization of recombinant lipases

#### Effect of pH and temperature

**Fig 4:**
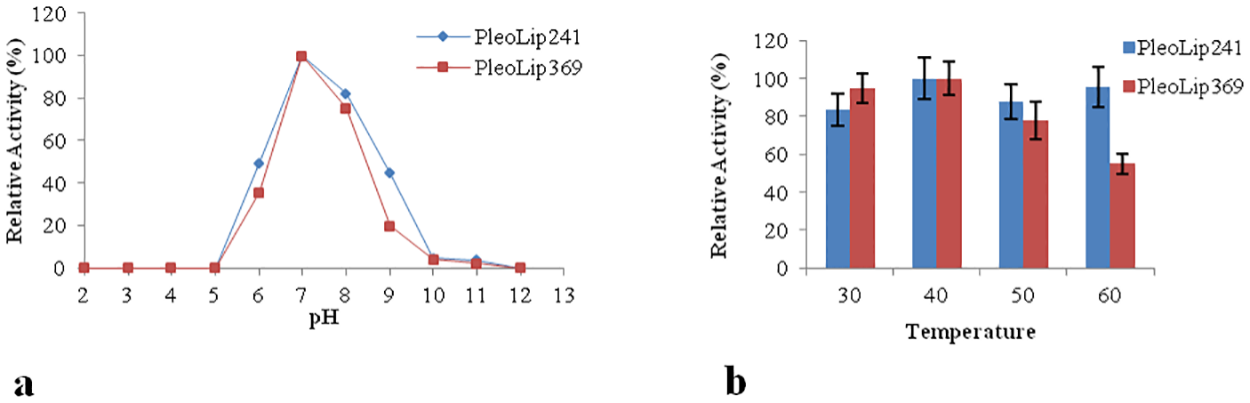
Effect of pH (a) and temperature (b) on PleoLip241 and PleoLip369 activities. The highest value of activity is considered as 100%. Standard deviations among three replicates were less than 5%.

Activity of both recombinant enzymes was tested in the pH range 2–12. Both enzymes were active between pH 6 and 9, with a maximum of activity at pH 7 (Fig 4a). Enzyme stability was assayed in the pH range 6–9 (Table 1). PleoLip241 showed a higher stability at all pH values in comparison with PleoLip369. Both proteins exhibited their optimal stability at pH 8.

**Table 1:** pH stability and thermostability of PleoLip241 and PleoLip369. Standard deviations were less than 5%.

	t _½_ (days)		t _½_ (days)		
pH	PleoLip241	PleoLip369	T (°C)	PleoLip241	PleoLip369
**6**	4	3	**4**	45	41
**7**	21	9	**30**	30	21
**8**	30	21	**40**	30	18
**9**	4	1	**50**	1	1
			**60**	0.04	0.04

The effect of temperature on the activity of both purified lipases was assessed in the range 30–60°C (Fig 4b). The two lipases retained the same activity level at 30°, 40°C and 50°C. However, while PleoLip241 maintained 100% activity also at 60°C, PleoLip369 activity was almost halved. Lipase stability was investigated in the same temperature range, and also at 4°C to assess protein storage stability (Table 1). Both enzymes were stable at 4°C with a t_1/2_ of about 40 days. PleuLip241 was found to be more stable than PleuLip369 at all the tested temperatures.

#### Effect of metal ions, detergents and organic solvents

The enzymatic activity of both lipases was tested in the presence of several ions, detergents and organic solvents (Table 2). PleoLip241 confirmed its higher stability respect to PleoLip369 in all the tested conditions.

**Table 2:** Effect of metal ions, solvents and detergents on PleoLip241 and PleoLip369 activities. Standard deviations were less than 5%.

	Residual activity (100%)			
	PleoLip241		PleoLip369	
**Metal ions**	**1 mM**	**10 mM**	**1 mM**	**10 mM**
NaCl	102	96	100	20
MgCl_2_	92	91	88	15
CaCl_2_	101	84	98	6
ZnCl_2_	83	37	86	0
KCl	88	83	55	7
CuSO_4_	50	0	35	0
FeSO_4_	50	20	27	0
MnCl_2_	100	95	77	18
**Solvents**	**10%**	**20%**	**10%**	**20%**
Ethanol	70	70	34	—
Glycerol	81	68	55	52
Aceton	45	40	20	—
Methanol	70	75	40	—
Methylacetate	65	50	—	—
t-Buthanol	80	65	62	35
Ethyl acetate	63	72	45	22
**Detergents**	**1%**		**1%**	
Tween20	0		0	
Tween 80	18		0	
Triton X100	135		115	
SDS	0		0	

The presence of Cu^2+^ and Fe^2+^ impaired the activity of both enzymes, conversely, an activity enhancement by these metals was reported by different authors [40,41].

In the presence of Tween 20, Tween 80 and SDS, lipase activity of both enzymes were remarkably inhibited. Conversely, Triton X-100 seemed to stabilize and improve both lipase activities, on the other hand examples of lipases inhibited by this detergent have been reported in the literature [42].

#### Substrate specificity

**Fig 5:**
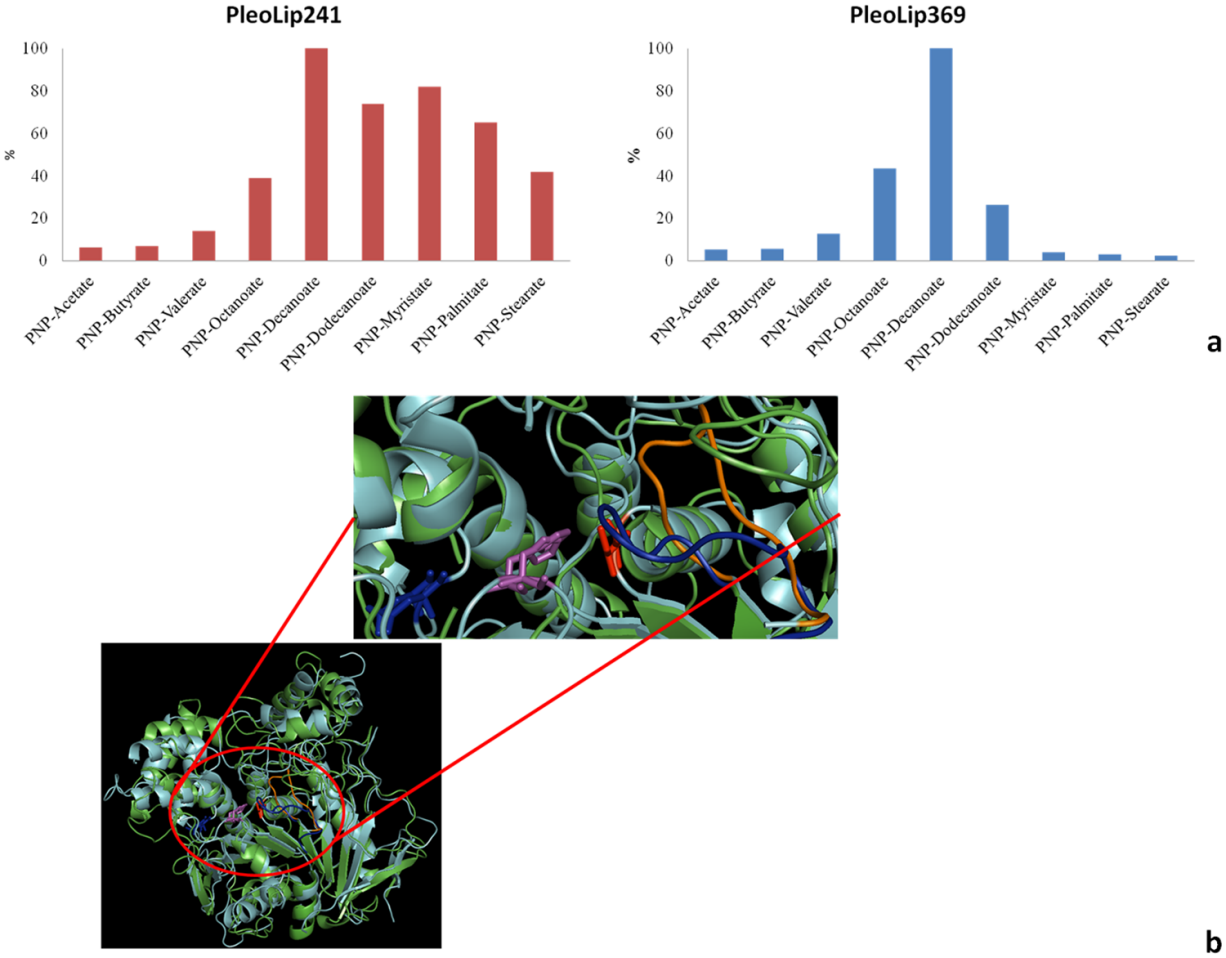
Substrate specificity of PleoLip241 and PleoLip369. **(a)** The specific activity of each lipase is reported as percentage of their respective activity towards the standard substrate (PNP-Decanoate). Standard deviations were less than 5%. **(b)** Overlapping of 3D models of PleoLip241 (cyan) and of PleoLip369 (green). Residues of the catalytic triad are displayed as sticks (Serine in red, Histidine in magenta, Glutammic acid in blue). The close-up highlights the loop (shown in blue) obstructing substrate pocket in PleoLip369. The corresponding region in PleoLip241 is shown in orange. Images were elaborated with *PyMol*.

Substrate specificity of both enzymes was assessed using substrates with different acyl chain length. PleoLip241 preferentially hydrolyzed long chains substrates with the following order C10>C14>C12>C16>C18≥C8, whereas PleoLip369 showed a narrower substrate specificity than PleoLip241 with preference towards C10 (Fig 5a). Based on these results, PleoLip241 can be classified as a true lipase. With the aim to understand the molecular reasons of the different substrate specificity displayed by the two enzymes, homology models of the two proteins were built. The modelled structures of both enzymes display the common α/β hydrolase fold of lipases and carboxylesterases (Fig 5b). Overlapping of protein models highlights an extra loop obstructing the substrate pocket accessibility in PleoLip369, probably responsible of its inability to hydrolize long chain substrates.

#### Kinetic parameters

Lipase kinetic parameters were assessed using their corresponding preferred substrates, *i*.*e*. Decanoate, Dodecanoate, Myristate and Palmitate for PleoLip241, and Octanoate, Decanoate, and Dodecanoate for PleoLip369 (Table 3). Both enzymes displayed very high affinity towards the tested substrates in comparison with other reported lipases [43–46]. PleoLip241 displayed a higher affinity and turnover towards both C10 and C12 acylic substrates respect to PleoLip369.

**Table 3:** Kinetic parameters of the recombinant lipases.

	PleoLip241		PleoLip369	
Substrate	K_M_ (μmol/L)	K_cat_ (min^-1^)	K_M_ (μmol/L)	K_cat_ (min^-1^)
**pNP-C8**	nd	nd	510 ± 60	0.11 ± 0.01
**pNP-C10**	30 ± 5	2.9 ± 0.4	130 ± 20	0.42 ± 0.05
**pNP-C12**	150 ± 6	1.6 ± 0.1	652 ± 75	0.21 ±0.02
**pNP-C14**	85 ± 4	1.2 ± 0.1	nd	nd
**pNP-C16**	350 ± 38	0.9 ± 0.1	nd	nd

### Effect of lipase pretreatment on the dyeability of wool

Considering the superior performances of PleoLip241 in terms of stability, specificity for long chain substrates and also the achieved production level, this enzyme was tested for its effect in improving wool dyeability. As a fact, wool displays on its surface a lipid layer that represents a hydrophobic barrier for dye uptake during the industrial process of colouring [47]. A lipase treatment can be a mild and eco-friendly alternative respect to the currently applied chemical methods affecting wool properties, and leading to an increase in environmental pollution. Pieces of pure new wool were treated with recombinant PleoLip241, and then dyed with a commercial dye. The dye remaining in the colouration bath after the dyeing process was measured. Comparison between the dyeing process of the untreated and the treated wool, revealed that a reduced amount of dye was found in the colouration bath of the lipase treated wool (55%) respect to the amount of dye found after colouration of the untreated sample (86%). It is possible to correlate this result with an increase of dyeability of wool after treatment with PleoLip241.

## Conclusions

A mining approach of the *P*. *ostreatus* genome revealed the presence of 53 putative lipase and 34 putative carboxylesterase coding-genes. Following a functional strategy, four of them were found to be differently expressed by *P*. *ostreatus* in the tested growth conditions. Two of the four identified proteins (PleoLip241 and PleoLip369), expressed in both tested conditions, were chosen for recombinant expression in *P*. *pastoris*. Despite their sequence similarity (58.9%), these enzymes exhibited dissimilar stability in all the investigated conditions and different substrate specificity. Indeed, by phylogenetic analysis the two proteins fall in different clusters.

Functional data allowed classifying PleoLip241 as true lipase, despite the automated annotation collocated it within the carboxylesterase family.

PleoLip241 was used to remove the hydrophobic layer from wool surface in order to improve its dyeability. The obtained results about wool dyeing encouraged the future exploitation of PleoLip241 in this field. As a fact, lipases can be applied not only to improve dyeability, but also to promote the uptake of different chemical compounds, such as anti-static, anti-perspirant or anti-microbial, in order to develop smart textiles.

## Supporting information

S1 Table Growth media conditions for extracellular lipase induction. The conditions that induce the production of extracellular lipase after five growth days are reported. All experiments have been conducted in triplicate.
(DOCX)

S1 Fig Multiple alignment among between lipase coding sequences from *P*. *ostreatus* and sequences of lipases from *Hypsizygus marmoreus* and from Pleurotus eryngii. In the grey box the conserved pentapeptide is displayed. The red arrows indicate the three aminoacids of catalytic triade.
(DOCX)

S2 Fig SDS-PAGE of recombinant lipases. Lane 1: Protein ladder; Lane 2: PleoLip369 crude extract; Lane 3: PleoLip241 crude extract; Lane 4: purified PleoLip369; Lane 5: purified PleoLip241.
(DOCX)
