# Supplementary material for: New lipases by mining of Pleurotus ostreatus genome
Source: PLoS One. 2017 Sep 25;12(9):e0185377. doi: 10.1371/journal.pone.0185377 (PMC5612753; doi:10.1371/journal.pone.0185377)
Supplement: S1 Table — The conditions that induce the production of extracellular lipase after five growth days are reported. All experiments have been conducted in triplicate. (DOCX) [file pone.0185377.s001.docx]

**S1 Table** Growth media conditions for extracellular lipase induction. The conditions that induce the production of extracellular lipase after five growth days are reported**.** All experiments have been conducted in triplicate.

| **Growth media** | **Activity halo** |
| --- | --- |
| PDY (basal condition) | - |
| PDY + 0.1% v/v olive oil | - |
| PDY + 0.5% v/v olive oil | - |
| PDY + 1% v/v olive oil | 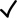 |
| PDY + 1% v/v OMW | - |
| PDY + 5% v/v OMW | 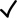 |
| PDY + 10% v/v OMW | 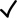 |
| PDY + 0.1% v/v glycerol | - |
| PDY + 0.5% v/v glycerol | - |
| PDY + 1% v/v glycerol | - |
| PDY + 5 g/L glucose | - |
| PDY + 7 g/L glucose | - |
| PDY + 10 g/L glucose | 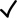 |
