# Supplementary material for: New lipases by mining of Pleurotus ostreatus genome
Source: PLoS One. 2017 Sep 25;12(9):e0185377. doi: 10.1371/journal.pone.0185377 (PMC5612753; doi:10.1371/journal.pone.0185377)
Supplement: S1 Fig — In the grey box the conserved pentapeptide is displayed. The red arrows indicate the three aminoacids of catalytic triade. (DOCX) [file pone.0185377.s002.docx]

369 MRFPSIFTAVLFAASSALAAPVNTTTEDETAQIPAEAVIGYLDLEGDFDVAVLPFSNSTN

*P.eryngii* --------------------------------------------------------MT--

241 ------------------------------------------------------------

*H.marmoreus* --------------------------------------------------------MAIL

369 NGLLFINTTIASIAAKEEGVSLDKRGNTTLYGRNLSDSGLEFYGGIPYAEPPLGDLRLRP

*P.eryngii* --FLLALSCLVSFTS---AAPQVQLGKTTLVGRDVTLLKQDFFGGVPFAEPPIGNLRLKP

241 ------------------------------------------------------------

*H.marmoreus* RGVATALALLPFIAA---QAPQVQVGNTAVIGRSIPEFGQELFGGIPFAEPPVGQLRLSN

369 PVPK-ALDVPSFDASDFGLMCYQRDLPAEVMSEDCLTINVLRPAGISSDASLPVMAWVHG

*P.eryngii*  PVLKTRLTQPAFDASNFGLACLQSGVPSGLISEDCLTINVFRPSGTAANASLPVLFWTYG

241 ------------------------------MSEDCLRINVLRPAGIPTGVVLPVMAWVYG

*H.marmoreus* PVLKTRLGTPTFDASNFGPACLQSAS-VPLMSEDCLRINVLRPAGIPTGVVLPVMAWVYG

:***** ***:**:* :.. ***: *.:*

369 GGFDQGSASEYNGTAIVAQSVARGTPVIYVNFNYRLGPLGFPQGREAAEKRALNVGLRDM

*P.eryngii* GGFDAGASAIYNGSAIVAQSVVRGTPLIYVNFNYRLGPLGFPQGQEADDRRALNLALKDQ

241 GGFDFGDSSIYNASAIVAQSVIRGTPVVFVSLNYRLGPLGFPQGVEAQKRGALNLGLKDQ

*H.marmoreus* GGFDFGDSSIYNASAIVAQSVIRGTPVVFVSLNYRLGPLGFPQGVEAQKRGALNLGLKDQ

**** * :: **.:******* ****:::*.:************ ** .: ***:.*:*

369 VLALNWIQDNIGVFGGDKAKVTVFGESAGAIALGTLMLGDTLDGLARAAIFQSGSAASTI

*P.eryngii* LAALEWVHQNIGLFGGDKNKVTVFGESAGAIMTAILFLNSPIQRLARGAIFESGSAGTAL

241 LAALEWVQANIGLFGGDKSKVTIFGQSAGSISLSIHFLNSNIKRLARAAIFESGFPATSL

*H.marmoreus* LAALEWVQANIGLFGGDKSKVTIFGQSAGSISLSILFLNSNIKRLARAAIFESGFTATSL

: **:*:: ***:***** ***:**:***:* . :*.. :. ***.***:** .:::

369 SVDTLDREADWHHFVTAIPACSMTAWTRDTFSCIRAADTSSLLPAVVAP--LALSKQIFP

*P.eryngii* TFNAARREINWQNFVSGVSRCASIATGGNTFGCLRAANSSDILQGLLKS--LADAPEKFG

241 NFPASHREQGWANFVKDVPQCASTAGSKDTFSCLRSDSIDEATLLKAASLADDQSGELFA

*H.marmoreus* NFPASHREQSWANFVKDVPQCASTAGSKDTFSCLRSDSIDEATLLKAGSLADDQSGELFA

.. : ** .* :**. : *: * :**.*:*: . .. : : *

369 WDNTIDGPGGFLPDLPSRLWERGLFAKIPFISGNNLDEGTLLTHPWVNSTEMLRETLIAN

*P.eryngii*  FDPTLDGPGGIFPDIPSKLFERGHFARLPFIAGTNLDEGTAFVPPSINASSQIRDSIIAN

241 WDPTIDGPGGILPDIPSKLLARGQFARLPFIAGTVLDEATTFTPKFITTEDQIRQSIIAN

*H.marmoreus* WDPTIDGPGGILPDIPSKLLARGQFARLPFIAGTVLDEGTTFTPKFITTEDQIRQSIIAN

:* *:*****::**:**:* ** **::***:*. ***.* :. :.: . :*:::***

369 YTPALMGERALNESVERLLELYPDVPALGSPFRTGNETFGLSSHFKRGCAILGDTIFHAQ

*P.eryngii* FSPPIVHPSLLENAADELLRLYPDDPVLGSPFNTGSETFGLSSVFKQASAIDGDINFQAQ

241 FTPSPFGPAVLAKTAETILQLYPDVPALGSPFGTGNETFGLSSQYKRAAAIFGDVSFQSQ

*H.marmoreus* FTPSPFGPAVLAKSAETILQLYPDVPALGSPFGTGNETFGLSSQYKRAAAIFGDVSFQSQ

::* . * ::.: :*.**** *.***** **.******* :*:..** ** *::*

369 RRKFSAVANRFGVKNWGYLFSDPPTTGPAFQGVAHLAELPYIFGTIDT-----PSYAKEL

*P.eryngii* RRFWMQTASKAGVKTFGYLFTEPQPNANPAVGVSHGSEVRFVYGQPE----NPTPSATRI

241 RRFWIQTLSKAGLKTFGYLFTDPQS-SDPVNGVSHASEIPYVYGAPGIFGGTVTPQALAL

*H.marmoreus* RRFWIQTLSKAGLKTFGYLFADPQS-SDPVNGVPHASEIPYVYGALGILGGTVTPQALAL

** : . .: *:*.:****::* . ** * :*: :::* * :

369 SSLMIDYWVSFATSLDPNDGKGLRRPVWPELTRRNQVLIEFIGNNTGVIPDNYRAEQIDF

*P.eryngii* SSIMIDYWVSFTTSLDPNDGRGIPRPIWSRYTPHNQVLMQLNGANLTLIPDDYRSEQIDF

241 SRIMVDYWVSFATSLDPNDGKGLPRPLWTQYTPSNQAIMLLNSTGTAMIPDDYRKKQIDF

*H.marmoreus* SRIMVDYWVSFATSLDPNDGKGLPRPLWTQYTPSNQAIMLLNSTGTTMIPDDYRKKQIDF

* :*:******:********:*: **:* . * **.:: : . . :***:** :****

369 IMGNLPVFQARH-----HHHHH

*P.eryngii* INSDPAVFHHRRSL--------

241 INSNPAVWHHRRSFSTHHHHHH

*H.marmoreus* INSNPAVWHHRRSFST------

* .: *:: *:

**S1 Figure.** Multiple alignment among between lipase coding sequences from *P. ostreatus* and sequences of lipases from *Hypsizygus marmoreus* and from [*Pleurotus eryngii*](https://it.wikipedia.org/wiki/Pleurotus_eryngii). In the grey box the conserved pentapeptide is displayed. The red arrows indicate the three aminoacids of catalytic triade.
