# Supplementary material for: New lipases by mining of Pleurotus ostreatus genome
Source: PLoS One. 2017 Sep 25;12(9):e0185377. doi: 10.1371/journal.pone.0185377 (PMC5612753; doi:10.1371/journal.pone.0185377)

**S2 Figure** SDS-PAGE of recombinant lipases. Lane 1: Protein ladder; Lane 2: PleoLip369 crude extract; Lane 3: PleoLip241 crude extract; Lane 4: purified PleoLip369; Lane 5: purified PleoLip241.


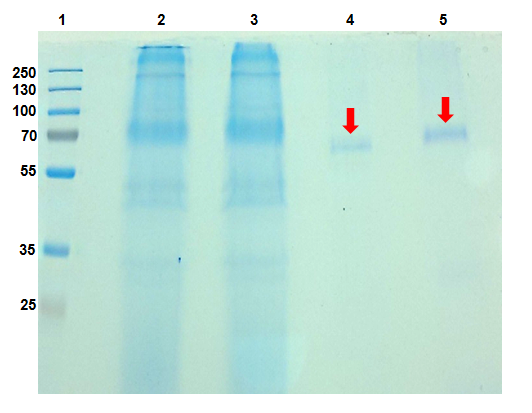

Supplement: S2 Fig — Lane 1: Protein ladder; Lane 2: PleoLip369 crude extract; Lane 3: PleoLip241 crude extract; Lane 4: purified PleoLip369; Lane 5: purified PleoLip241. (DOCX) [file pone.0185377.s003.docx]
